# The Perceived Availability of Online Social Support: Exploring the Contributions of Illness and Rural Identities in Adults with Chronic Respiratory Illness

**DOI:** 10.3390/ijerph17010242

**Published:** 2019-12-29

**Authors:** Samantha R. Paige, Rachel E. Damiani, Elizabeth Flood-Grady, Janice L. Krieger, Michael Stellefson

**Affiliations:** 1 STEM Translational Communication Center, University of Florida, Gainesville, FL 32611, USA; rdamiani@ufl.edu (R.E.D.); efloodgrady@ufl.edu (E.F.-G.); janicekrieger@ufl.edu (J.L.K.); 2 Clinical and Translational Sciences Institute, University of Florida, Gainesville, FL 32610, USA; 3 Department of Health Education and Promotion, East Carolina University, Greenville, NC 27858, USA; stellefsonm17@ecu.edu

**Keywords:** online social support, social identity, communication theory of identity, rural health, chronic obstructive pulmonary disease

## Abstract

Joining an online social support group may increase perceived membership to a community, but it does not guarantee that the community will be available when it is needed. This is especially relevant for adults with Chronic Obstructive Pulmonary Disease (COPD), many of whom reside in rural regions and continually negotiate their illness identity. Drawing from social support literature and communication theory of identity, this cross-sectional study explored how COPD illness and geographic identities interact to influence patients’ perceived availability of online social support. In April 2018, 575 adults with a history of respiratory symptoms completed an online survey. Patients with a COPD diagnosis reported greater availability of online support. This was partially mediated by a positive degree of COPD illness identity (i.e., being diagnosed with COPD, a history of tobacco use, severe respiratory symptoms, high disease knowledge, and low income but high education). The relationship between COPD illness identity and the availability of online support was strongest among those with low rural identity; however, at lower levels of COPD illness identity, participants with high rural identity reported the greatest degree of available online support. Results have important implications for tailored education approaches across the COPD care continuum by illness and geographic identities.

## 1. Introduction

The perceived availability of social support has a positive effect on health behaviors and associated outcomes [1,2,3], including stress reduction and improved quality of life [4]. Social support is an interpersonal communication process characterized by the exchange of informational and emotional resources among and across networks [5,6]. Identifying and engaging social networks is a prerequisite to achieve associated benefits of social support; however, a significant value of social support lies within the perception that others will be available, should the need for support arise [7].

The Internet transcends lifespan and geographic boundaries to increase users’ access to social support. Social support groups exist online and through social media to help patients cope with a particular health condition or risk behavior. These programs, which are intended to supplement traditional offline support, provide patients with emotional and informational resources [8]. Unlike face-to-face support transactions, however, these technology mediated programs reduce the social and contextual cues used to reduce uncertainty and thereby better address informational and communicative needs [9]. The rising number of online support programs and their increased following is a testament for their growing interest among individuals affected by chronic disease [8,10]. A remaining concern is the degree to which patients believe the illness management support will be available when they need it.

Membership in online disease-specific social support programs constitute a “rite of passage” for patients who are diagnosed with a chronic condition [11]. However, there is a degree of social belonging that needs to occur for an individual to sufficiently benefit from online support. In general, people begin to identify as a member of an online social network by learning, negotiating, and adopting the values and attitudes of others [12]. These shared values and beliefs increase the likelihood of engaging in behaviors consistent with group norms [12]. As described by the Communication Theory of Identity (CTI) [13,14], a disconnect between group membership and identity often results in adverse health behaviors and poor communicative outcomes, which can reduce the availability of social support. As such, receiving a diagnosis and joining an online support program may increase perceived membership to a social network (i.e., social identity), but it does not guarantee that the network will be available for support when it is needed.

A unique disease context to study the theoretical intersection between perceived availability of online social support and social identity is Chronic Obstructive Pulmonary Disease (COPD), which is a progressive lower respiratory condition that predominantly affects rural adults over the age of 45 with a history of smoking tobacco [15]. Because COPD is a progressive condition, patients must engage in timely, on-going self-management of symptoms [16]. The self-management of COPD is considered a communal- rather than intrapersonal-level endeavor [17], as it is optimized through care coordination with multiple stakeholders in COPD decision-making. Physical, psychosocial, and social support are noted as critical for patients with COPD to thrive with their condition. Social and psychosocial support include (but are not limited to) emotional regulation, developing relationships and supplementing offline support, as well as navigating services and information about maintaining independence [18]. Although there has been a recent call for expanding access to online social support in COPD [19], these patients are generally older and report challenges when navigating online communities [20]. Therefore, there is a need to understand the social identities that may contribute to the perceived availability of online social support [21], especially among patients who live in isolated rural regions where health disparities persist yet access to the Internet increases.

### 1.1. Perceived Online Social Support

Social support is broadly conceptualized as “verbal and nonverbal communication between recipients and providers that reduces uncertainty about the situation, the self, the other, or the relationship and functions to enhance a perception of personal control in one’s experience” [5] (p. 19). Social support can be perceived (i.e., belief that social support will be available) or received (i.e., exchanged or provided) [22]. An abundance of literature highlights the value of perceived social support in enhancing health promotion and reducing mortality in diverse patient populations [23,24]. For instance, there is evidence supporting the importance of perceptions of an available social network among patients with COPD. The presence of a supportive person or network (e.g., simply residing with a supportive person) is associated with positive health behaviors, including smoking cessation and participation in pulmonary rehabilitation [25]. As such, the perceived availability of social support can generally have a positive effect on patients to enhance their quality of life [26].

There is growing interest in how Internet and social media use impacts patients with COPD [27]. Social media, particularly platforms that provide support for chronic illnesses (e.g., COPD), extend patients’ access to informational and emotional resources enabling them to better cope with their illness [8]. This is likely why patients with chronic disease more often participate in online social support than those without a chronic condition [28]. Research exploring the potential of online support groups for COPD patient education has focused on the quality and modality of various self-management topics communicated by diverse sources, including peers and professional organizations [29,30]. Patients with COPD also actively use social media to disclose symptoms (e.g., cough, mucus and sputum, shortness of breath) and seek patient education resources [31]. Martinez and colleagues [32] support this finding, indicating that patients with COPD more frequently (at least weekly) use the Internet if they experience severe symptoms and complications. Nevertheless, these patients generally reside in a rural region and have limited access to health care insurance. As such, the Internet is used as a health care supplement to obtain new information and seek second opinions about a health status or recommendation.

Online social support groups are “any virtual social space where people come together to get and give information or support, to learn, or to find company” [33] (p. 348). The primary focus of research examining the potential of the Internet to facilitate social support among COPD patients has been geared toward its receipt rather than its provision. Giving and receiving social support represents two different acts to achieve communication and behavior-oriented goals. Reblin and Uchino [22] systematically reviewed literature on social support and health-related implications, reporting the need to better understand the mechanisms by which people are motivated to both give and receive social support. These authors posit that that people who give social support may be motivated not only by informational needs, but also to “feel good” about themselves or feel valued by giving back to the community. And while there is evidence that people who receive social support are likely to reciprocate it, there is limited evidence to demonstrate whether or not a diagnosis of COPD contributes to how the availability of online social support is perceived among these patients. Accordingly, this study tested the following hypothesis:

**Hypothesis** **1:** *A positive relationship exists between a diagnosis of COPD and perceived availability of online sources to (a) give and (b) receive social support.*

### 1.2. Social Identities: Illness and Geographic

Much like social support, the social identities of people are continuously constructed and validated, through interactions with others who may be within and outside of a particular social network. As such, communication is a core element of social identity formation and adaptation [13]. Social identities are cultivated when cultural underpinnings of a particular illness or region are integrated into a person’s self-concept to explain the beliefs, values, and behaviors of its members [34]. One type of social identity is illness identity, comprising a set of roles and attitudes that a person develops in relation to their understanding of having a disease or disorder [35]. Simply being diagnosed with a condition does not guarantee that an individual relates with others diagnosed or living with the condition. This phenomenon is extremely relevant to the context of COPD, where smoking tobacco is a primary and stigmatized behavioral risk factor for most people living with the condition, yet there is also a predisposing genetic variant that causes the disease for some who have never smoked tobacco in their lifetime [15]. Knowledge about COPD is suboptimal and considerably conflicted [26], which may affect the degree to which a patient identifies with the condition and others who have been diagnosed. Mass media campaigns (e.g., Tips from Former Smokers© [36]) advertise the “face of COPD” as an older adult, typically a male with a history of heavy smoking tobacco who exhibits respiratory distress that necessitates use of supplemental oxygen [37]. On the contrary, pharmaceutical companies advertise treatments for COPD by highlighting symptoms (e.g., the metaphorical elephant on chest) experienced by middle-to-older age adults who value enjoying time with family. These ads rarely mention risk behaviors (e.g., tobacco use) or equipment (e.g., oxygen tanks) that elicit public stigma. Although these campaigns raise awareness of COPD, they evoke differential emotions (e.g., fear, disgust, hope) to consequently shape the public’s view of COPD. To date, however, limited empirical attention has examined—from the perspective of patients diagnosed with COPD—which experiences, symptoms, and demographic characteristics are most influential in shaping this illness identity. Therefore, this study aims to answer the following research question:

**RQ 1:** *What factors are reported as comprising the chronic respiratory illness identity?*

Those with a strong social identity hold beliefs and engage in behaviors that enhance the image of their group membership to make them distinct from other competing groups [12]. Likewise, people who belong to a particular community, or network, but do not strongly identify with their membership are likely to deviate from established group norms [12]. Because social support is a communicative, coping phenomenon that promotes the health and well-being of its recipients, social identities should, theoretically, function in a manner that is conducive to enhancing the availability of social support. Given widespread adoption of the Internet and its variety of social support forums for COPD [29,31,38,39], it is expected that patients with a strong illness identity will report positive perceptions about available online social support. This study tested the following hypothesis:

**Hypothesis**  **2:** *COPD illness identity will positively mediate the relationship between receiving a diagnosis of COPD and perceived availability of online social support.*

Another type of social identity is geographic identity, defined as the aspects of one’s self that are derived from the culture and natural environment of a physical location [40]. One salient facet of geographic identity includes the traits, values, and behaviors that emerge from residing in either a rural or urban location. Rural adults, for example, engage in riskier health behaviors (e.g., heavy tobacco use, fewer preventive efforts) and report less trust in government safety and regulatory bodies [41]. Although there are sub-cultures that are apparent in rural areas, the rural culture is generally associated with prioritizing relationships with family [42], and satisfaction with living in the same community for long periods of time without any desire to leave [43]. Consequently, rural adults are more likely to value strongly rooted social networks, particularly those comprised of familiar others (e.g., neighbors) who hold similar cultural beliefs. Given this cultural profile, individuals with a high degree of rural identity may be more likely to receive informational support in online groups comprised of users with similar characteristics. This is unlike those who have a low rural identity, who may be more likely to become immersed in the online experience to exchange support with the intention of building new relationships with others who have dissimilar values. Because rural adults are disproportionally affected by COPD [44] and hold unique cultural values about health and social networks, empirical attention is needed to examine how illness and rural identities interact to affect the perceived availability of online social support. Therefore, this study aims to answer the following research question:

**RQ 2:** *Does rural identity moderate the mediating effect of COPD illness identity on the perceived availability of online social support?*

## 2. Materials and Methods

In April 2018, 575 adults participated in a 20-min web-based survey. Patients from a university research registry with chronic lower respiratory conditions (ICD-10 Codes J40-J47), minus J45 “asthma”), were enrolled in the study through a combination of United States Postal Service (USPS) mailer and email notifications (*n* = 283). Participants were also invited to participate through a publicly accessible university-based research listing website, where a survey description and link were advertised as a “lung health study” (*n* = 292).

### 2.1. Measures

**Socio-demographics.** Items from the Behavioral Risk Factor Surveillance System [45] were used to capture socio-demographic features of the sample. This included age (in years), gender, race, ethnicity, as well as income and education.

**COPD diagnosis and identity.** Participants were asked whether they have been diagnosed with COPD (1 = Yes; 0 = No). To assess COPD illness identity, participants were asked the degree to which they identified with other patients who live with COPD (1 = fully disagree; 7 = fully agree). This item captured communal identity related to COPD, which differs from existing illness identity measures that assess the degree to which an illness is integrated into an individual’s self-identity [46].

**COPD knowledge.** COPD knowledge was measured with Maples and colleagues’ [47] 13-item COPD-Questionnaire (COPD-Q), including items about etiology, pathophysiology, management, and symptoms. Each literacy-sensitive item included three nominal response options (True, False, Not Sure). Correct responses were coded as 1, whereas items answered incorrectly or uncertain by the participant were coded as 0. A summative score was computed for analysis (0 = low knowledge; 13 = high knowledge).

**COPD symptom severity.** Hallmark COPD symptoms include dyspnea, persistent cough, chest congestion, and fatigue [15]. Reflecting these symptoms, four items were created to assess the degree that these symptoms were present in the participant’s life. Each item was anchored on a 5-point Likert-type scale (1 = strongly disagree; 5 = strongly agree). Responses were aggregated and averaged (α = 0.70). A score of 0 indicated the absence of symptoms, whereas a 5 indicated a strong symptom presence.

**Tobacco use.** An item from the Behavioral Risk Factor Surveillance System [45] measured tobacco use. First, participants were asked how long it had been since they last smoked a cigarette. Respondents who selected “15 years or more” or “I have never smoked regularly” were categorized as not recent/current smokers. This cutoff was selected because 15 years after quitting smoking puts an individual at an equal risk of cardiovascular disease as someone who has never smoked [48]. Also, a former 30 pack-year smoker is no longer eligible for lung cancer screening, a common tobacco-associated lung condition, if they quit smoking 15 years ago or more [49]. All other responses were coded as current or recent tobacco users.

**Rural location and identity.** Perceived rural residence was measured by asking, “to what extent would you describe your current location (where you live)?” Perceived rural identity was measured by asking, “to what extent do you identify as being from a small town/rural or urban/city? Both items were scored based on a 5-point Likert-type scale (1 = city/urban; 5 = small town/rural).

**Perceived availability of online social support.** The Shakespeare-Finch and Obst [4] 2-Way Social Support Scale was adapted to assess how people reciprocate health-related online social support using a 5-point Likert-type scale (1 = not at all; 5 = always). The original instrument used to measure reciprocal social support was comprised of four scales, which distinguished between emotional and instrumental support. We added language to reflect online social support for the purposes of adaptation. Preliminary analyses found that data produced by these two support styles were highly correlated for the perceived availability of online sources to receive and give support (*r* = 0.86; *p* < 0.001). For the purposes of parsimony, the four scales were collapsed to measure two constructs: perceived availability of sources to (a) give (10 items; α = 0.93) and (b) receive (10 items; α = 0.94) online social support. Each scale’s average score was computed for analyses.

### 2.2. Data Analysis

SPSS v25 (IBM, Armonk, NY, USA) was used to conduct all statistical analyses, which handled missing data by applying list-wise deletion procedures. Frequency statistics were computed to describe the sample. To answer RQ1, a hierarchical linear regression analysis, with attention to *R*^2^ change, was conducted to examine how socio-demographics, COPD diagnosis and risk factors, and COPD knowledge contributed to COPD illness identity scores. Hayes’ PROCESS v3.1 macro was used to test Hypotheses 1–3. Data were fit to Model 15 to carry out two conditional process analyses. Model 15 tests a moderated mediation with two moderations occurring between (a) the independent variable and dependent variable and (b) the mediated variable and dependent variable. This model examines the indirect effect of a COPD diagnosis (independent variable; IV) on the perceived availability of online social support (to give and to receive; dependent variables; DVs) via COPD illness identity differences (Mediator), conditional on level of rural identity (Moderator). For the first interaction effect, the product of COPD status and rural identity was produced to determine the moderation effect on perceived availability of online support (DV 1 = give; DV 2 = receive). For the second interaction, the product of COPD illness identity and rural identity was mean-centered. The Johnson-Neyman technique was used to probe for interactions at different levels of the moderator. Data were normally distributed; therefore, the moderator was collapsed into three levels: −1*SD*, *M*, +1*SD* [50]. PROCESS v3.1 macro uses boot strapping was conducted to generate 95% confidence intervals.

## 3. Results

Table 1 shows the socio-demographic, COPD-related, and online activities of participants. Participants were, on average, 55 years old and predominantly white, although nearly 20% identified as Hispanic. Over half earned more than $50,000 annually and nearly 80% reported at least some college education.

Perceived residence and rural identity were moderately rural. The Mdn for each item was 3, which is similar to the M (SD) values reported in Table 1. About 10% of participants perceived their residence as extremely (value = 1 on the 5-point Likert-type scaled item) urban/metropolitan (*n* = 66; 11.7%) and reported the lowest possible degree of rural identity (*n* = 53 or 9.3%). Conversely, nearly 20% perceived their residence as extremely (value = 5 on the 5-point Likert-type scaled item) small/town rural (*n* = 103; 18.3%) and reported the highest degree of rural identity (*n* = 100; 17.5%). A Pearson’s r correlation demonstrates that perceived residence and geographic identity has a positive, but not perfect correlation (r = 0.74; *p* < 0.001).

Despite nearly 71% of participants reporting a COPD diagnosis and moderately severe symptoms, participants’ COPD illness identity was average (M = 3.16; SD = 1.77), on a 7-point Likert-type scale. Less than half (42.1%) reported as a current smoker or a smoker who quit within the past 15 years. A greater proportion of participants reported never smoking (*n* = 208; 36.3%), as compared to those who had smoked in the past but quit more than 15 years ago (*n* = 123; 21.5%). Participants also reported an average degree of COPD knowledge (M = 6.45; SD = 2.74).

Participants perceived a moderate degree of online source availability to give and receive support. Although approximately 20% of the sample reported not having any available online support to receive (*n* = 110, 19.5%) or give (*n* = 132; 23.5%) health information, over 60% of participants reported using social media at least five hours each week.

### 3.1. Research Question 1

Table 2 shows that socio-demographics, COPD diagnosis and risk factors, and COPD knowledge contribute to COPD illness identity. In regard to socio-demographics, only lower income and higher education was associated with a greater COPD illness identity, *F* (8, 467) = 3.86, *p* < 0.001 (*R^2^* = 0.06, *R*^2^_adj_ = 0.05). While controlling for socio-demographics, having a self-reported COPD diagnosis, reporting more severe respiratory symptoms, and identifying as a current/recent smoker were all positively associated with COPD illness identity, *F* (11, 464) = 13.17, *p* < 0.001 (*R^2^* = 0.24, *R*^2^_adj_ = 0.22). Finally, COPD knowledge was positively associated with COPD illness identity, *F* (12, 463) = 16.40, *p* < 0.001 (*R^2^* = 0.30, *R*^2^_adj_ = 0.28).

### 3.2. Hypotheses 1–2

Table 3 presents results of conditional process analyses, supporting both H1 and H2. Both models show that factors contributing to COPD illness identity were statistically significant for opportunities to give, *F* (501, 8) = 20.15, *p* < 0.001 (*R^2^* = 0.24), and receive online support, *F* (504, 8) = 21.05, *p* < 0.001 (*R^2^* = 0.25). As expected, a COPD diagnosis had a positive, statistically significant association with COPD illness identity in both models (*p* < 0.001). When factors from Table 2 were entered into the same regression step, socioeconomic status and identifying as a current/former tobacco smoker were no longer statistically significant predictors of COPD illness identity.

**Figure 1:**
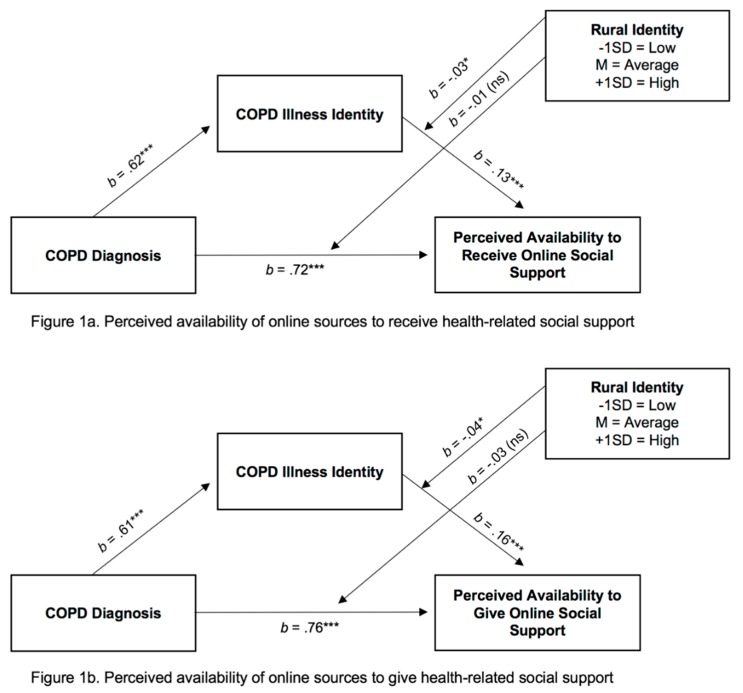
Conceptual models depicting moderated indirect effects. (**a**) Perceived availability of online sources to receive health-related social support; (**b**) Perceived availability of online sources to give health-related social support. Note. * p < 0.05; *** *p* < 0.001; ns = not statistically significant (*p* > 0.05).

Table 3 also demonstrates the cumulative models examining COPD status, COPD illness and rural identities while controlling for covariates of perceived availability of online sources to give, *F* (497, 12) = 39.57, *p* < 0.001 (*R^2^* = 0.49), and receive support, *F* (500, 12) = 37.50, *p* < 0.001 (*R^2^* = 0.47). Figure 1 shows that COPD diagnosis (*b* = 0.76 and 0.72, respectively) and COPD illness identity (*b* = 0.16 and 0.13, respectively) had a positive, statistically significant association with perceived availability of online sources for which they could give and receive support. In regard to covariates, being younger, residing in a less rural area, and reporting lower COPD knowledge was associated with greater perceived availability of these support sources.

### 3.3. Research Question 2

**Figure 2:**
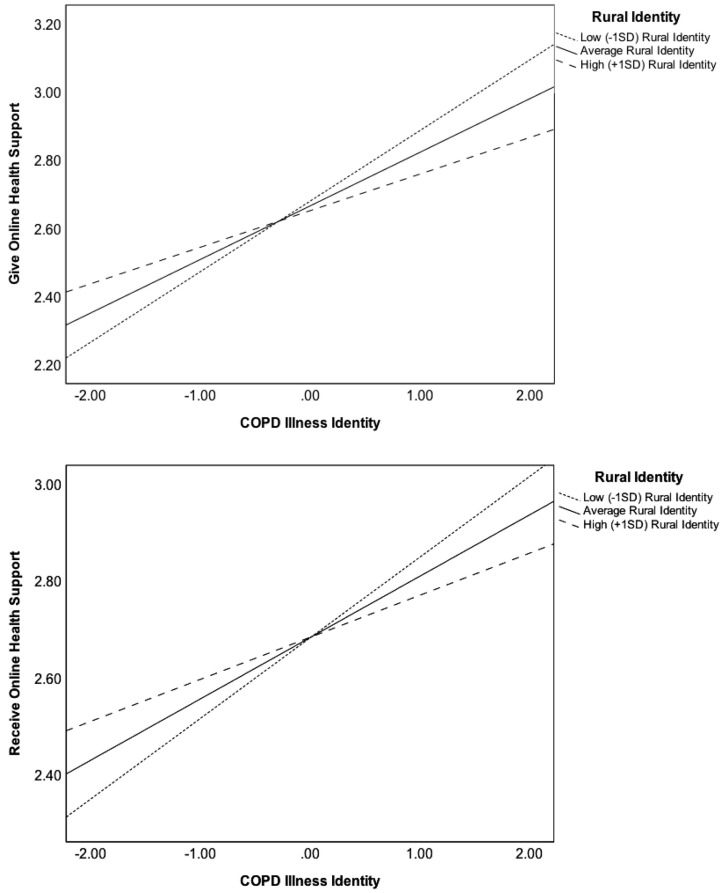
Moderating effect of rural identity on illness identity and reciprocating online support.

Table 3 further demonstrates that rural identity moderates the relationship between COPD illness identity and the perceived availability of online sources to give (*b* = −0.04) and receive (*b* = −0.03) support (*p* < 0.05). Figure 2 depicts the moderation effect. Johnson-Neyman analyses indicated that the interaction strength was strongest for low rural identity (effect = 0.24; standard error = 0.04) and weaker for high rural identity (effect = 0.07; standard error = 0.04). Rural identity did not moderate the direct relationship between COPD diagnosis and perceived availability of online social support.

Table 4 demonstrates the direct effect of a COPD diagnosis on the perceived availability of online social support and the indirect effect through COPD illness identity at three levels of rural identity (−1*SD*, *M*, +1*SD*). A partial mediation exists. The non-statistically significant index of moderated mediation indicated that this indirect relationship is independent of rural identity as a moderator.

## 4. Discussion

### Principal Findings

This study explored how social identities, including illness and rurality, contributed to perceptions of available online sources for giving and receiving social support among patients with a history of respiratory health conditions. Reporting a COPD diagnosis was positively associated with COPD illness identity, as well as perceived availability of online sources for social support. Consistent with social identity and communication literature [13], respondents with a strong COPD illness identity also reported having online sources available for social support. Results also demonstrate that the relationship between COPD illness identity and perceived availability of online social support was strongest among participants with low rural identity. Findings have important implications for considering the interplay of multiple layers of social identity in understanding online social support in COPD.

Socio-demographics, specifically low income and high education, were attributed to COPD illness identity; however, COPD experiences (i.e., having a physician COPD diagnosis, identifying as a current/recent smoker) and symptoms (i.e., reporting more severe respiratory symptoms) explained the greatest amount of variance in identity. Unexpectedly, results demonstrated that neither geographic region, gender, race/ethnicity, nor age was associated with COPD illness identity. These findings underscore the outdated stereotype of COPD as an older white man’s disease [37].

Consistent with research that chronic disease populations are more likely than their non-diagnosed counterparts to engage in online media activities [28], self-reporting a COPD diagnosis was positively associated with perceived availability of being able to give and receive online social support. This relationship was partially mediated by COPD illness identity. Interestingly, a greater perception of online social support existed among participants with COPD, who were younger and had low COPD knowledge. Although there is evidence that greater respiratory symptom severity predicts patients’ Internet use [27,32], research has demonstrated that high COPD knowledge and less severe symptoms are associated with a higher degree of eHealth literacy (i.e., ability to access, understand, and evaluate online information) [27]. Patients who are at a critical point of illness identity negotiation may not be able to maximize the potential of online social support, because they do not have the optimal skills to do so. Future research is needed to understand how patients navigate these online experiences with varying degrees of knowledge and symptoms.

Rural identity did not moderate the relationship between having a COPD diagnosis and reporting available online support; however, it did interact with COPD illness identity, a partial mediator that explained the relationship between COPD diagnosis and perceived available online support. Results demonstrated that the positive relationship between COPD illness identity and perceived available online support was strongest among individuals who reported low rural identity. Despite these results, there is evidence that online chronic disease interventions are both effective and accepted in rural regions to promote disease-specific knowledge and psychosocial adaptations [51]. We further examined this relationship and found that patients with a high rural identity reported a greater degree of online social support than their counterparts with low rural identity, but only when COPD illness identity was low. This provides a unique opportunity to promote the early detection of COPD among high-risk rural adults by targeting patients with health education that helps build their communal identity with COPD. Conversely, for urban adults with high COPD illness identity, online interventions that integrate social support may be more effective later in the COPD care continuum, or immediately after a diagnosis when the adoption of new self-management behaviors becomes imperative. Future research is needed to understand the timing and amount of online support interventions across the geographic and care continuums in COPD. This will provide support in culturally adapted respiratory health communication interventions that consider the intersection of illness and geographic identities.

This study examines how two communal identities (i.e., COPD illness and rural) interact to explain how patients with COPD reciprocate online support to cultivate community ties. Although geographic region and culture may not function as part of an individual’s COPD illness identity, these social identities have a synergistic effect on perceptions of available online social support. Central to theoretical underpinnings of the CTI [13], identity “gaps” or discrepancies between actual and perceived group membership can lead to poor communicative outcomes. Consistent with this theory, the relationship between COPD risk factors and perceptions of illness identity were more favorable to the perceived availability of online social support for health-related purposes. Reporting an urban residence was positively associated with greater availability of online social support; however, low rural (or urban) identity did not significantly moderate the relationship between COPD status and perceived availability of online social support. The only instance where rural identity moderated the relationship between diagnosis and perceived availability of online social support was in conjunction with a positive COPD illness identity. In future social support research conducted through the theoretical lens of social identity and CTI, identity should be examined as a dynamic and multi-faceted construct that influences perceptions of online social support. Rather than isolate one component of identity (e.g., illness identity), findings emphasize the need to adopt a holistic perspective to understand health behaviors by considering multiple components of communal identity simultaneously. 

The perceived availability to give and receive online social support was highly correlated in this study, indicating that this sample comprising predominantly of patients with COPD believe they have support systems on the Internet with whom they can exchange or reciprocate health information. This provides support for the theoretical argument that a person who receives support is likely to reciprocate it [22]. These scales are expected to correlate to some degree [4]; however, we did not expect the association to be so strong in this patient population, which is why we separated the two scales to examine predictors of perceived availability to give and to receive online support. Empirical evidence supports that patients with COPD face challenges navigating online platforms simply to access and evaluate health content from informational websites, and only about 30% of patients with COPD use social media (e.g., Facebook) [27]. In the current study, however, about 60% of participants were active on social media more than 5 h a week, equivalent to nearly one hour each day. It is unclear from this study whether frequent use of social media resulted in a greater degree of perceived availability of online support, or whether being directed to online support forums by a family member, peer, or health care provider resulted in greater use of these media. Regardless, this finding justifies national efforts to increase COPD patients’ access and use to online communities [19], which are shown to improve self-management behaviors and health-related outcomes [25,26].

This study does not exist without limitations. A single item measured illness and geographic identities, as valid and reliable measures of these constructs either do not exist or are limited in their theoretical scope. Finally, this was a cross-sectional study, meaning associations are correlative not causative. This presents a potential limitation but highlights an important direction for future research. For example, we aimed to understand what factors are associated with COPD illness identity in this study, and one of those factors was COPD knowledge. It is possible that a person with a high COPD illness identity may be naturally more inclined to seek more information about COPD, thus being more knowledgeable. The potential for reverse causality in the relationship between COPD illness identity and COPD knowledge brings attention to the importance of more experimental and longitudinal research in understanding how illness identities manifest across the care continuum (e.g., screening, diagnosis, and across treatment). Likewise, research is needed to examine the interaction of geographic and illness identities in other health-related contexts, particularly those that are acute and not progressive.

## 5. Conclusions

This study examines elements of social identity through a communication lens to understand how patients perceive the availability of online health-related social support. Results provide evidence that illness and geographic identities contribute to the perceived availability of online sources of social support in the context of COPD. Further, results facilitate an understanding for the point on the COPD care continuum that rural/urban patients may be most receptive to online social support. Receptivity will be likely in these two contexts: (1) high rural identity coupled with low COPD illness identity, and (2) low rural identity coupled with high COPD illness identity. For rural communities, in particular, social media facilitates opportunities for instrumental and emotional social support that can help adults with respiratory distress recognize the burden of their disease and prompt early detection of exacerbated symptoms.

## Figures and Tables

**Table 1:** Characteristics of the sample (*n* = 575).

Predictor	Descriptive Statistics
Socio-Demographics	
Age, *M* (*SD*)	55.14 (12.62)
Gender, *n* (%)	
Female	256 (44.50)
Male	298 (51.80)
Missing	21 (3.70)
Race, *n* (%)	
White	474 (82.4)
Black/African American	54 (9.4)
Asian American	12 (2.1)
Native American	13 (2.3)
Other/Multi-Racial	19 (3.3)
Missing	1 (0.7)
Ethnicity, *n* (%)	
Hispanic	108 (18.8)
Non-Hispanic	433 (75.3)
Missing	34 (5.9)
Income, *n* (%)	
$24,999 or less	70 (12.2)
$25K–$34,999	60 (10.4)
$35K–$49,999	124 (21.6)
$50K–$74,999	147 (25.6)
$75K or more	156 (27.1)
Missing	18 (3.1)
Education, *n* (%)	
Less than High School	37 (12.7)
High School or Equivalent	66 (11.5)
College 1–3 Years	180 (31.3)
College 4 or More Years	288 (50.1)
Missing	4 (0.7)
**Chronic Obstructive Pulmonary Disease (COPD)-Specific Factors**	
COPD Illness Identity, *M* (*SD*)	3.16 (1.77)
Respiratory Symptom Severity, *M* (*SD*)	2.81 (0.96)
Disease Knowledge, *M* (*SD*)	6.45 (2.74)
Smoker (Current, Quit within 15 years), *n* (%)	242 (42.2)
Reported COPD Diagnosis, *n* (%)	407 (70.8)
**Rural-Specific Factors**	
Rural Identity, *M* (*SD*)	3.01 (1.22)
Perceived Rural Residence, *M* (*SD*)	2.98 (1.29)
**Online Activity and Support Behaviors**	
Give Online Health Support, *M* (*SD*)	2.57 (1.15)
Receive Online Health Support, *M* (*SD*)	2.58 (1.16)
Weekly Social Media Use, *n* (%)	
0–1 h	119 (20.7)
2–4 h	99 (17.2)
5–9 h	63 (11)
10+ h	289 (50.3)
Missing	5 (0.9)

**Table 2:** Hierarchical linear regression of factors contributing to COPD illness identity.

				95% CI	
Regression Steps	*R*^2^ Change	*b*	*SE b*	Lower Bound	Upper Bound
**Step 1: Socio-Demographics**	0.05 **				
Age		−0.01	0.01	−0.02	0.01
Gender ^a^		−0.25	0.15	−0.56	0.05
Race ^b^		0.31	0.23	−0.15	0.77
Ethnicity ^c^		0.37	0.22	−0.05	0.79
Income ^d^		−0.60 **	0.16	−0.91	−0.29
Education ^e^		0.57 *	0.23	0.12	1.03
Rural Identity		0.06	0.10	−0.13	0.25
Perceived Rural Residence		−0.02	0.10	−0.21	0.16
**Step 2: COPD Experiences**	0.22 **				
COPD Diagnosis		0.79 **	0.19	0.42	1.15
Respiratory Symptoms		0.54 **	0.09	0.37	0.71
Smoker ^f^		0.32 *	0.15	0.01	0.62
**Step 3: COPD Awareness**	0.28 **				
COPD Knowledge		0.18 **	0.03	0.12	0.23

Note. CI = Confidence Interval; ^a^ Gender (1 = Female; 0 = Male); ^b^ Race (1 = White; 0 = Non-White); ^c^ Ethnicity (1 = Hispanic; 0 = Non-Hispanic); ^d^ Income (1 = $50K or more/year; 0 = $49,999 or less/year); ^e^ Education (1 = At least some college; 0 = High school or less); ^f^ Smoker (Current or quit within the past 15 years). * p < 0.05; ** p < 0.01.

**Table 3:** Conditional process analyses of identity on online health-related engagement.

	Regression Models, *b* (*SE*) [95% CI]	
Regression Steps	Outcome 1: Perceived Availability to Give Online Support	Outcome 2: Perceived Availability to Receive Online Support
**Step 1: COPD Illness Identity**		
*n*(*df*)	501(8)	504(8)
*F* Statistic	20.15 ***	21.05 ***
*R*^2^ Value	0.24	0.25
COPD Status	0.61 (0.17) [0.28, 0.95] ***	0.62 (0.17) [0.29, 0.96] ***
Symptom Severity	0.55 (0.08) [0.39, 0.71] ***	0.54 (0.08) [0.38, 0.69] ***
COPD Knowledge	0.17 (0.03) [0.11, 0.22] ***	0.17 (0.03) [0.12, 0.22] ***
Smoker	0.12 (0.15) [−0.16, 0.41]	0.11 (0.15) [−0.18, 0.40]
Perceived Rural Residence	−0.05 (0.06) [−0.02, 0.01]	−0.05 (0.05) [−0.15, 0.06]
Age	−0.01 (0.01) [−0.02, 0.01]	−0.01 (0.01) [−0.02, 0.01]
Income	−0.24 (0.14) [−0.52, 0.03]	−0.24 (0.14) [−0.51, 0.03]
Education	0.40 (0.19) [0.04, 0.77] *	0.35 (0.19) [−0.02, 0.71]
**Step 2: Online Support Outcome**		
*n*(*df*)	497(12)	500(12)
*F* Statistic	39.57 ***	37.50 ***
*R*^2^ Value	0.49	0.47
COPD Status	0.76 (0.09) [0.57, 0.94] ***	0.72 (0.10) [0.52, 0.91] ***
COPD Illness Identity	0.16 (0.02) [0.11, 0.21] ***	0.13 (0.02) [0.08, 0.18] ***
Rural Identity	−0.01 (0.04) [−0.10, 0.07]	0.01 (0.05) [−0.09, 0.09]
*COPD Status* * Rural Identity	−0.03 (0.07) [−0.16, 0.11]	−0.01 (0.07) [−0.15, 0.12]
*COPD Identity* * Rural Identity	−0.04 (0.02) [−0.07, −0.01] *	−0.03 (0.02) [−0.07, 0.00] *
Symptom Severity	−0.05 (0.04) [−0.14, 0.04]	−0.03 (0.04) [−0.12, 0.06]
COPD Knowledge	−0.04 (0.02) [−0.07, −0.01] **	−0.06 (0.02) [−0.09, −0.03] ***
Smoker ^a^	0.07 (0.08) [−0.08, 0.23]	0.04 (0.08) [−0.12, 0.20]
Perceived Rural Residence	−0.22 (0.04) [−0.30, −0.13] ***	−0.19 (0.04) [−0.27, −0.10] ***
Age	−0.03 (0.01) [−0.04, −0.03] ***	−0.04 (0.01) [−0.04, −0.03] ***
Income	−0.07 (0.08) [−0.21, 0.08]	−0.14 (0.07) [−0.29, 0.01]
Education	0.16 (0.10) [−0.04, 0.37]	0.19 (0.10) [−0.01, 0.40]

Note. CI = Confidence Interval; ^a^ Smoker (Current/quit within the past 15 years); * p < 0.05; ** p < 0.01; *** p < 0.001.

**Table 4:** Direct and indirect effects by geographic identity (−1*SD*, *M*, +1*SD*).

		95% Confidence Interval	
Direct/Indirect Pathway	Effect (Std. Error)	Lower Level	Upper Level
**Direct Effect** COPD Diagnosis -> Give Online Support			
Low Rural Identity	0.79 (0.13)	0.54	1.05
Average Rural Identity	0.76 (0.09)	0.57	0.94
High Rural Identity	0.72 (0.12)	0.49	0.96
COPD Diagnosis -> Receive Online Support			
Low Rural Identity	0.74 (0.13)	0.48	1.00
Average Rural Identity	0.72 (0.10)	0.53	0.91
High Rural Identity	0.71 (0.12)	0.47	0.94
**Indirect Effect** COPD Diagnosis -> COPD Illness Identity -> Give Online Support			
Low Rural Identity	0.13 (0.05)	0.04	0.24
Average Rural Identity	0.10 (0.04)	0.03	0.18
High Rural Identity	0.07 (0.03)	0.02	0.13
COPD Diagnosis –> COPD Illness Identity -> Receive Online Support			
Low Rural Identity	0.10 (0.04)	0.03	0.20
Average Rural Identity	0.08 (0.03)	0.02	0.15
High Rural Identity	0.05 (0.03)	0.01	0.12

Note. Low rural identity (−1SD) = −1.20; Average rural identity (M) = 0.0; High rural identity (+1SD) = 1.20.
